# An Attention-Based Multidimensional Fault Information Sharing Framework for Bearing Fault Diagnosis

**DOI:** 10.3390/s25010224

**Published:** 2025-01-03

**Authors:** Yunjin Hu, Qingsheng Xie, Xudong Yang, Hai Yang, Yizong Zhang

**Affiliations:** 1 Key Laboratory of Advanced Manufacturing Technology of the Ministry of Education, Guizhou University, Guiyang 550028, China; 17785011779@163.com; 2 School of Mechanical Engineering, Guizhou University, Guiyang 550028, China; xdyang@gzu.edu.cn (X.Y.); haiykeep@gmail.com (H.Y.); yizongzhang1@163.com (Y.Z.)

**Keywords:** small samples, fault diagnosis, public knowledge, training weights

## Abstract

Deep learning has performed well in feature extraction and pattern recognition and has been widely studied in the field of fault diagnosis. However, in practical engineering applications, the lack of sample size limits the potential of deep learning in fault diagnosis. Moreover, in engineering practice, it is usually necessary to obtain multidimensional fault information (such as fault localization and quantification), while current methods mostly only provide single-dimensional information. Aiming at the above problems, this paper proposes an Attention-based Multidimensional Fault Information Sharing (AMFIS) framework, which aims to overcome the difficulties of multidimensional bearing fault diagnosis in a small sample environment. Specifically, firstly, a shared network is designed to capture the common knowledge of the Fault Localization Task (FLT) and the Fault Quantification Task (FQT) and save it to the global feature pool. Secondly, two branching networks for performing FLT and FQT were constructed, and an attentional mechanism (AM) was used to filter out features from the shared network that were more relevant to the task to enhance the branching network’s capability under small samples. Meanwhile, we propose an innovative Dynamic Adjustment Strategy (DAS) designed to adaptively regulate the training weights of FLT and FQT tasks to achieve optimal training results. Finally, extensive experiments are conducted in two cases to verify the effectiveness and superiority of AMFIS.

## 1. Introduction

As a key component of mechanical equipment, rolling bearings are extensively used in modern industrial machines. However, during the continuous operation of the equipment, the poor working environments will cause damage or deterioration to the rolling bearings, which will lead to mechanical failure, bring economic losses to enterprises, and even endanger personal safety [1]. Work [2] discussed the diagnosis and root causes of bearing faults. The author points out that over 40% of motor failures are caused by bearing faults, and by implementing fault diagnosis and maintenance procedures, the reliability and stability of rotating machinery can be significantly improved [3,4].

Deep learning has excellent abilities in feature extraction and pattern recognition [5], which have led to the wide application and rapid development of intelligent fault diagnosis techniques based on deep learning in the field of fault diagnosis. For example, Fu et al. [6] designed a composite network architecture that fuses a convolutional neural network (CNN) and a long short-term memory network (LSTM). This model is capable of feature extraction of bearing vibration signals from both time series and spatial distribution dimensions simultaneously, aiming to enhance the accuracy of feature extraction and the stability of the model. Li et al. [7] proposed a reinforcement integration method for diagnosing rolling bearing faults under different operating conditions, selecting the optimal base learner by designing a reinforcement model and constructing an integration model using a sparse ANN, and the experimental results show that this method is superior to other intelligent methods and has significant value for engineering applications, but it requires larger computational resources and time. Lei et al. [8] developed an advanced intelligent bearing fault diagnosis technique that incorporates the adaptive variational modal decomposition technique. This method can adaptively process non-stationary vibration signals, decompose them into multiple instantaneous frequency components, and further select a series of effective frequency components for real-time online fault diagnosis. Jia et al. [9] improved the accuracy and efficiency of fault diagnosis by fusing temporal and spatial features. The method demonstrated superior performance in experiments, especially in terms of classification accuracy. However, the data volume factor needs to be considered in practical applications.

Although deep learning-based fault diagnosis techniques have shown certain advantages in practical applications, they still face two main challenges: First, engineering practice typically requires fault information from multiple dimensions (e.g., fault detection, localization, and quantification), whereas most current deep learning models can only provide fault results in a single dimension. Second, fault data in real engineering is often limited, and deep learning models are prone to overfitting with insufficient training data, which can affect the accuracy of fault diagnosis. The small sample problem is particularly prominent in practical engineering applications [10,11,12,13,14] because the collected fault data are often limited, especially in the fault diagnosis of certain critical or new equipment. In this case, how to use the limited data to construct a model that can accurately diagnose multiple fault types is an urgent problem [15]. Meanwhile, due to the complexity of faults, the diagnostic information from a single perspective is often insufficient to comprehensively assess the fault state [16], and thus, there is an increasing demand for multidimensional fault diagnosis.

Multi-task learning (MTL), a learning method that collaboratively processes multiple tasks, shows potential to address the aforementioned challenges. This approach is able to capture common features of multiple related tasks simultaneously and share information across tasks, thus improving the generalization of the model [17]. In the framework of MTL, through the flow of information during the training process, individual tasks can indirectly absorb beneficial data from other tasks, alleviating the dependence of traditional deep learning models on large-scale datasets [18,19]. Currently, although MTL has shown its strong potential, research that simultaneously considers small sample problems and multidimensional fault information sharing has yielded relatively limited results so far. In literature [20], an adaptive MTL-based bearing fault diagnosis framework was proposed, which combines data augmentation, image coding techniques, and convolutional neural networks to achieve high-precision fault type and severity classification. Despite the use of data augmentation, the model performance is still highly dependent on the quality and quantity of training data, and the lack of diverse data may limit the model’s generalization ability. Literature [16] proposed a deep multi-task, multi-scale information fusion method based on MTL for fault type diagnosis and fault size localization but did not validate its performance in a small sample scenario. The literature [21] demonstrated high diagnostic accuracy and efficiency by simultaneously identifying the bearing fault location and severity through a deep residual network, with the advantage of using residual learning to solve the problem of gradient vanishing in deep network training and enhancing the generalization ability of the model through multi-scale feature extraction. In addition, the literature [22] proposes a new approach based on multi-task conditional generation of adversarial networks to address the challenges of bearing fault diagnosis under small sample sizes and variable operating conditions. Overall, there have been studies that have achieved initial results in the areas of small-sample learning and multidimensional fault diagnosis, but they usually do not combine these two elements for comprehensive consideration. These research approaches limit the models to have large limitations in practical applications, as real-world fault diagnosis scenarios often need to deal with both sample scarcity and multidimensional fault information [23,24].

To address the shortcomings of existing research, this paper proposes an Attention-based Multidimensional Fault Information Sharing (AMFIS) framework aimed at overcoming the challenges of multidimensional bearing fault diagnosis in a small-sample environment. The architecture is able to process the Fault Localization Task (FLT) and Fault Quantification Task (FQT) tasks of rolling bearings in parallel, which enhances the performance and generalization ability of the model under data scarcity through the multidimensional fault information sharing and attention allocation mechanism. Specifically, a shared network is first designed to capture the public knowledge of both FLT and FQT tasks and save it to the global feature pool. This design ensures that the model effectively exploits the key features common to both tasks, laying the foundation for subsequent specialized task processing. Next, two branching networks for performing FLT and FQT were constructed, and an Attention Mechanism (AM) was used to filter out the more task-relevant features from the shared network to enhance the branching network’s capability under small samples. Finally, to ensure that both FLT and FQT tasks can achieve the optimal training effect, this paper proposes an innovative, dynamic weight adjustment strategy which can adaptively adjust the training weights of FLT and FQT tasks according to the training process so as to achieve more effective task optimization.

The contribution of this paper relative to existing work is summarized below:

(1) Focusing on the challenges of multidimensional bearing fault diagnosis in small sample environments, a multidimensional fault information-sharing architecture based on the AM is proposed.
(2) In order to ensure that both FLT and FQT tasks can achieve optimal training results, this paper proposes an innovative, dynamic weight adjustment strategy, which can adaptively adjust the training weights of FLT and FQT tasks according to the training process in order to achieve more effective task optimization.
(3) By conducting extensive experiments in two cases, the effectiveness and superiority of AMFIS are verified, providing a feasible solution path for small-sample multidimensional bearing fault diagnosis.

## 2. Relevant Theories

### 2.1. Multi-Task Learning

When exploring new skills, people often rely on the knowledge and skills they have already accumulated. In the example of learning Taekwondo, those who are already proficient in Taekwondo tend to adapt more quickly when transitioning to martial arts. MTL is similar in that the generalization of the model can be enhanced through knowledge integration and experience transfer across tasks [25,26]. Compared to training a single task independently, MTL focuses more on the interaction and synergy between tasks, aiming to improve the learning efficiency and prediction accuracy of the model [27].

In AMFIS, we set up two tasks, FLT and FQT, which have the same input samples $x^{i}$ but different task labels $y_{flt}^{i}$ and $y_{fqt}^{i}$, and the dataset is defined as follows:

(1) $$D={\left\{x^{i},y_{flt}^{i},y_{fqt}^{i}\right\}}_{i}^{m}$$

where *i* denotes the *i*-th sample.

### 2.2. Attention Mechanism

AM is an important technique in the field of deep learning, inspired by human visual attention, allowing models to focus on the most important parts of the present while processing information [28]. Originally studied in the fields of neuroscience and psychology, this mechanism was later introduced into machine learning and has been widely used, especially in the fields of Natural Language Processing (NLP) [29] and Computer Vision (CV) [30]. The basic attention model can be expressed as:

(2) $$\hat{X}=\alpha \cdot X$$

where α is the attention weight and X and $\hat{X}$ are the original and weighted features, respectively.

In recent years, a series of AM module design solutions have emerged, including the Squeeze-and-Excitation (SE) module [31], Convolutional Block Attention Module (CBAM) [32], Efficient Channel Attention (ECA) Module [33], etc. Currently, AM is also widely used in the field of intelligent fault diagnosis of machinery to improve recognition performance. For example, the AM introduced in the works [34,35,36] can help perform more accurate feature extraction and thus improve the performance of bearing fault detection.

## 3. Proposed Method

**Figure 1:**
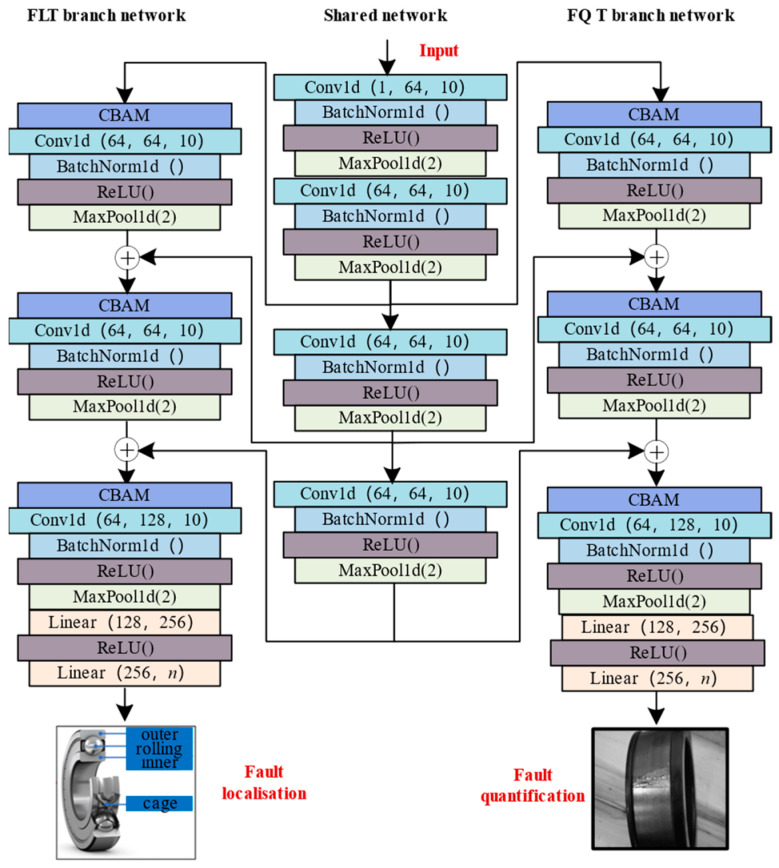
Details of the AMFIS architecture.

In this section, the details of AMFIS are described in detail. The network architecture of AMFIS is shown in Figure 1, which mainly consists of a shared network, FLT branch network, and FQT branch network. The data in parentheses represent the important parameters of the layer. For example, in Conv1d (64, 64, 10), the numbers in parentheses represent the number of input channels, the number of output channels, and the size of the convolution kernel, respectively.

### 3.1. CBAM Attention Module

In this section, we first introduce the Attention module, and in the next section, we will focus on the detailed operation of AMFIS.

The shared network captures public features from different tasks; however, these features need to be further processed to serve a specific task. To make the public features captured by the shared network serve a specific task effectively, we design several CBAM modules, which are able to further refine these public features, identify and highlight the features that are more important for a specific task while suppressing the less relevant or redundant information to better adapt to the needs of each specific task. Specifically, the CBAM module has two main attention mechanisms: channel attention and spatial attention. The channel attention mechanism evaluates the importance of different channels, reinforcing those feature channels that contribute to the task while suppressing those that are less important. The spatial attention mechanism, on the other hand, focuses on the importance of different locations on the feature map, highlighting those regions that contain critical information.

**Figure 2:**
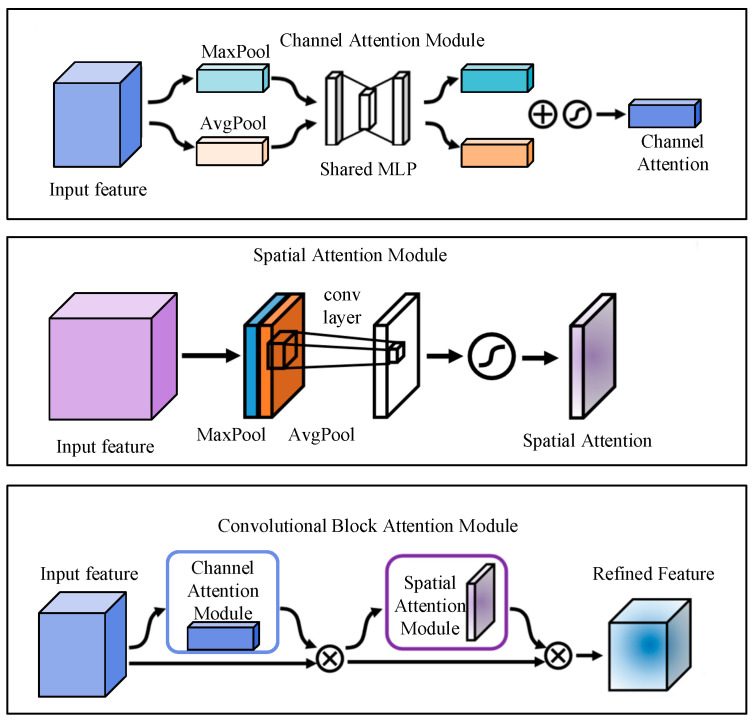
CBAM Attention Module.

The design of CBAM is shown in Figure 2, which consists of a channel attention module and a spatial attention module. The main idea is to integrate the spatial and channel attention in order to enhance the feature representation of the model.

Firstly, the channel attention module generates weights for each channel, indicating its importance. For the input feature map $F\in R^{H\times W\times C}$, the global average aggregation is performed for each channel, and the global maximum is taken for each channel, respectively.

(3) $$F_{avg}=\frac{1}{H\times W}\sum_{i=1}^{H}\sum_{j=1}^{W}F_{i,j,c}$$

(4) $$F_{max}=\underset{i=1,\ldots ,H;j=1,\ldots ,W}{max}F_{i,j,c}$$

Equations (2) and (3) yield two feature vectors $F_{avg}$ and $F_{max}$ of length *C*. $F_{avg}$ and $F_{max}$ are each passed through a shared multilayer perceptron (MLP), which consists of two fully connected layers activated in the middle using the ReLU:

(5) $$M_{\mathrm{avg}}=\mathrm{MLP}\left(F_{\mathrm{avg}}\right)$$

(6) $$M_{max}=\mathrm{MLP}\left(F_{max}\right)$$

The weights of each channel are obtained by summing $M_{\mathrm{avg}}$ and $M_{max}$ with Sigmoid activation:

(7) $$\alpha_{c}=\sigma \left(M_{\mathrm{avg}}+M_{max}\right)$$

where σ is the Sigmoid function and $\alpha_{c}$ is the channel attention.

Weight each channel of the input feature map:

(8) $$F^{\prime }=\alpha_{c}\cdot F$$

Second, spatial attention generates weights for each location, indicating the importance of each spatial location. For the input $F^{\prime }\in R^{H\times W\times C}$, the channel dimensions of $F^{\prime }$ are aggregated using average pooling and maximum pooling to obtain two feature maps $F_{\mathrm{avg}}^{\prime }$ and $F_{max}^{\prime }$ of shape H×W:

(9) $$F_{\mathrm{avg}}^{\prime }=\frac{1}{C}\overset{C}{\underset{c=1}{\sum }}F_{i,j,c}^{\prime }$$

(10) $$F_{max}^{\prime }=\underset{c=1,\ldots ,C}{max}F_{i,j,c}^{\prime }$$

Further $F_{\mathrm{avg}}^{\prime }$ and $F_{max}^{\prime }$ are spliced in the channel dimension to form a feature map of shape H×W×2:

(11) $$F^{\beta }=\mathrm{Concat}\left(F_{\mathrm{avg}}^{\prime },F_{max}^{\prime }\right)$$

A 7 × 7 convolution is applied to $F^{\beta }$ to generate a single-channel spatial weight map β:

(12) $$\beta =\sigma \left({\mathrm{Conv}}_{7\times 7}\left(F^{\beta }\right)\right)$$

Finally, each position is weighted to get the final output $F^{\prime \prime }$:

(13) $$F^{\prime \prime }=\beta \cdot F^{\prime }$$

### 3.2. AMFIS Operation Process

The AMFIS operation process is shown in Figure 1. Firstly, the data sample $D={\left\{x^{i}\right\}}_{1}^{n}$ is fed into the shared network for feature extraction, and the feature extraction process of convolutional block can be expressed as follows:

(14) $$x_{s}^{l}=Maxpool\left(Relu\left(BN\left(\overset{C^{n-1}}{\underset{i=1}{\sum }};k_{i,c}^{n}\times x_{i}^{n-1}+b_{c}^{n}\right)\right)\right)$$

where $k_{i,c}^{n}\in R^{W\times 1}$ refers to the kernel connecting the channels of layer *n* − 1 to the channels of layer *n*; $C^{n-1}$ denotes the number of channels of layer *n* − 1; $x_{i}^{n-1}$ denotes the output of the channels of layer *n*; $b_{c}^{n}$ is the bias value. Relu denotes the Relu activation function, Maxpool denotes the maximum pooling process, BN denotes the batch normalization; $x_{s}^{l}$ denotes the output of the *l*-th convolutional block of the shared network.

Next, the output $x_{s}^{2}$ of the 2nd convolutional block is fed into the next convolutional module of the shared network, FLT, and FQT branch networks, respectively, as shown in Figure 1. In the branch network, the CBAM module weights $x_{s}^{2}$ according to Equations (3)–(13) to obtain the features related to the respective tasks. Meanwhile, the branching network performs feature extraction again according to Equation (14) to obtain the task features $x_{t}^{1}$.

Finally, the shared feature $x_{s}^{3}$ extracted by the next convolutional module of the shared network is fused with the task feature $x_{t}^{1}$ extracted by the branching network as shown in Equation (15):

(15) $$x=x_{s}^{3}+x_{t}^{1}$$

where x represents the fused features, $x_{s}^{3}$ denotes the shared features extracted from the 3rd convolutional block of the shared network, and $x_{t}^{1}$ denotes the task features extracted from the 1st convolutional block of the branch network.

Throughout the process, a total of two feature fusions were performed, and the process is similar, so it will not be repeated.

### 3.3. AMFIS Model Optimization

Under the AMFIS framework, the model needs to handle multiple parallel tasks simultaneously and optimize them jointly. However, given the differences in the difficulty and data size of each task, this may lead to rapid mastery of some tasks while others are difficult to achieve the desired results, resulting in the problem of over-learning of easy-to-learn tasks while difficult-to-learn tasks are difficult to converge.

To cope with the problem of uneven learning results due to varying task difficulty in multi-task learning, this paper proposes a DAS. The strategy aims to balance the optimization efforts of the FLT and FQT to ensure that the learning process can proceed adequately when faced with tasks of varying difficulty. The DAS evaluates the learning progress of the current task by monitoring the change of the loss function of each task during the training process and accordingly assigns higher weights to the tasks with higher difficulty in order to promote their learning efficiency and effectiveness.

First, the loss $L_{i}$ for each task is calculated as a percentage of the current moment.

(16) $${\hat{L}}_{i}=\frac{L_{i}+\epsilon }{\sum_{j=1}^{N}\left(L_{j}+\epsilon \right)}$$

where $L_{i}$ is the loss of the *i*-th task and ϵ is a smoothing factor to prevent the weights from collapsing as the task loss approaches 0.

Secondly, Softmax weights are calculated based on ${\hat{L}}_{i}$.

(17) $$w_{i}\left(t\right)=\frac{2\cdot \exp\left(-\alpha \cdot {\hat{L}}_{i}\right)}{\sum_{j=1}^{N}\exp\left(-\alpha \cdot {\hat{L}}_{j}\right)}$$

where α controls the sensitivity of weight changes to losses. Larger α amplifies the weight variance and defaults to 0.1. $w_{i}\left(t\right)$ is the weight of the *i*-th task at moment *t*.

Both FLT and FQT belong to classification tasks, so the cross entropy is chosen as the objective function, and the total loss function is:

(18) $$L_{\mathrm{total}}=\overset{N}{\underset{i=1}{\sum }}w_{i}\left(t\right)\cdot \left(-\overset{m}{\underset{k=1}{\sum }}y_{i}^{k}\left(\log{\hat{y}}_{i}^{k}\right)\right)$$

where N denotes the total number of tasks, m denotes the total number of samples, k denotes the k-th sample, $y^{k}$ is the true value and ${\hat{y}}^{k}$ is the predicted value.

## 4. Experiments and Discussion

In this section, the effectiveness of the proposed method will be verified through experiments on two case datasets.

### 4.1. Experimental Details

All experiments in this paper are NVIDIA GTX1080Ti GPU (manufactured by Colorful, located in Shenzhen, China) and Pytorch 1.40, with batch size and learning rate set to 32 and 0.01, respectively, and a training Epoch of 200. It should be noted that (1) all experiments in this paper are repeated 5 times and averaged. (2) All single-task learning methods train and test FLT and FQT separately, and MTL methods train FLT and FQT jointly.

### 4.2. Comparison Methods

To verify the superiority of AMFIS, SVM, WDCNN [37], Siamese networks [11], HSMTL [21] and Cross-stitch [38] are compared in Table 1.

### 4.3. Case 1: Western Reserve University Dataset

#### 4.3.1. Data Presentation and Processing

**Figure 3:**
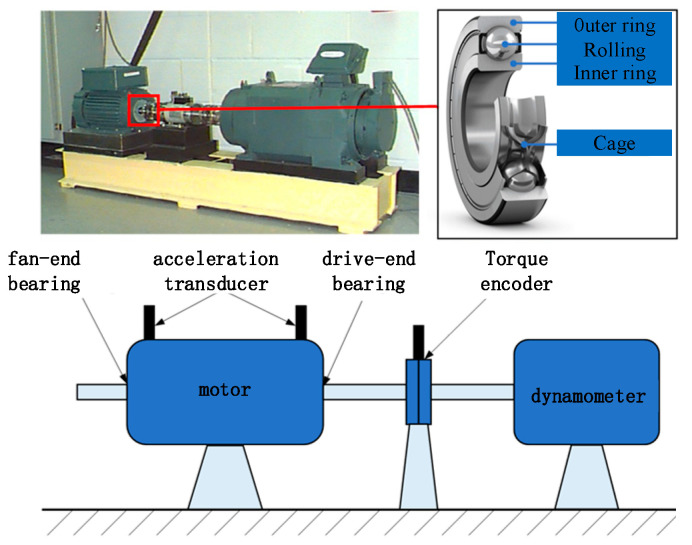
CWRU data acquisition platform.

**Figure 4:**
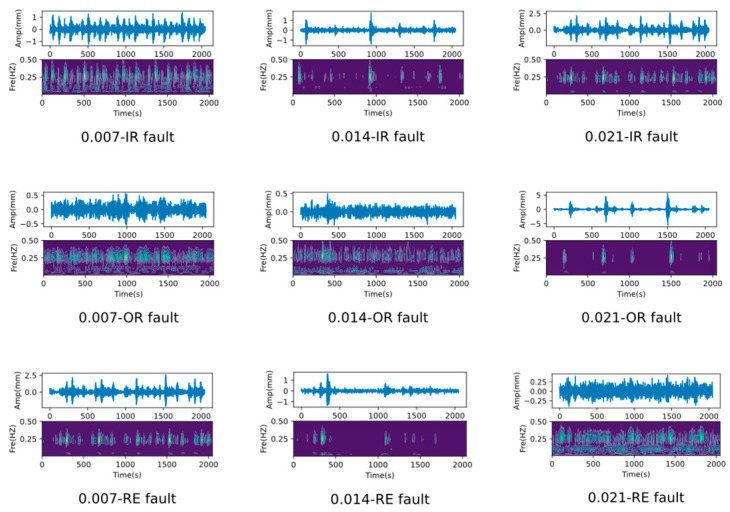
Visualization of the time-frequency diagram for different faults.

We first used the Western Reserve University (CWRU) dataset to validate the proposed methodology. The data acquisition platform is shown in Figure 3, where the fault vibration data were recorded for motors operating at 0 hp, 1 hp, 2 hp, and 3 hp loads, respectively. All the bearing failures were single-point damaged by Electric Discharge Machining (EDM), and a total of three types of failures were simulated, i.e., Outer Ring (OR) failure, Inner Ring (IR) failure, and Rolling Element (RE) failure, as well as three types of damage level failures of 0.007, 0.014, and 0.021 inches, and the vibration signals time-frequency plots of these failures are demonstrated in Figure 4.

**Figure 5:**
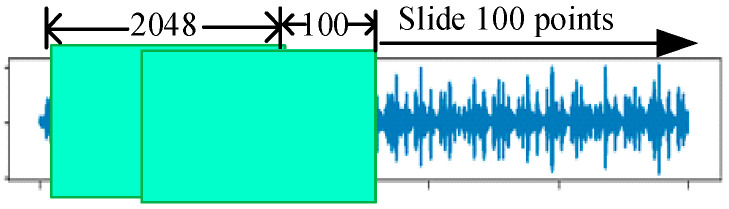
Fault sample handling process.

In this paper, the main experiments are carried out using drive end-bearing data with a sampling frequency of 12 kHz. The sample processing is shown in Figure 5, and a resampling method is adopted; the length of each fault sample is 2048 data points, and the sliding window is 100. Finally, the details of the fault samples obtained are shown in Table 2.

#### 4.3.2. Performance Validation of AMFIS with Small Samples

In this section, we focus on evaluating the small-sample fault diagnosis performance of the proposed method under 0 to 3 hp load conditions. Specifically, we randomly selected 60 samples from 0 to 3 hp loads for training while using 2000 samples for testing. The experimental results are summarized in Table 3, where the bolded numbers represent the best performance, while the underlined numbers indicate the sub-optimal performance.

As can be seen in Table 3, the performance of different methods varied under different loading conditions. For example, under 0 hp conditions, AMFIS exhibits the highest accuracy on both FLT and FQT tasks, with 88.13% and 87.63%, respectively. While under 1 hp conditions, Siamese performs best on FQT with an accuracy of 84.43%.

Taken together, soft-shared MTL methods (e.g., Cross-stitch and AMFIS) outperform other traditional methods in small-sample fault diagnosis scenarios overall. This result suggests that the knowledge-sharing strategy in multi-task learning is effective. With a limited number of samples, knowledge sharing between different tasks can complement and enrich the knowledge required for fault diagnosis, thus improving the diagnostic capability of the model. The Siamese network’s few-shot learning mechanism for small-sample fault diagnosis does achieve good results on some tasks, but performance varies significantly between tasks. This may be due to the fact that the Siamese network relies on a similarity metric for learning, which may be more likely to capture similarities between samples for some tasks and may not be sensitive enough for others. Unsurprisingly, SVM has the worst performance because it relies on finding the optimal decision boundary in the feature space, which is difficult to achieve with a limited number of samples. In contrast, AMFIS achieves knowledge enrichment by effectively integrating fault information from both tasks, enabling AMFIS to demonstrate significant performance advantages in fault diagnosis tasks under different load conditions.

**Figure 6:**
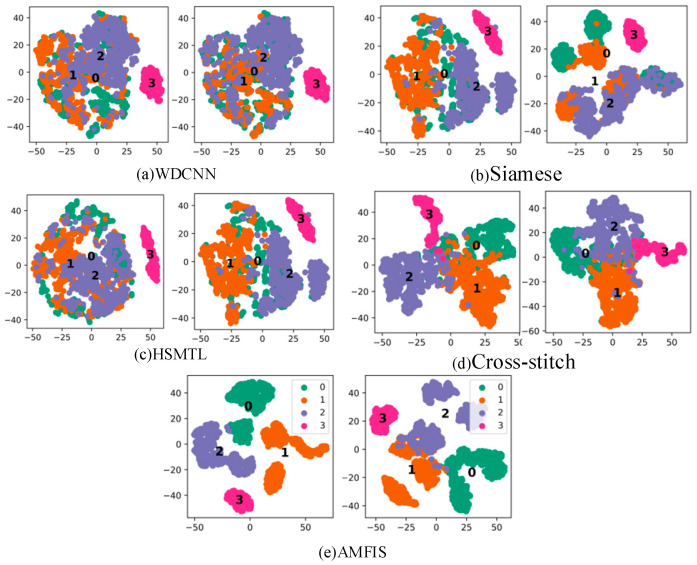
Output layer feature T-SNE visualization. The left image of each set is FLT, and the right image is FQT.

In order to explore the performance of different methods in depth, we performed T-SNE visualization of the features extracted by all methods under 3 hp conditions, as shown in Figure 6.

**Figure 7:**
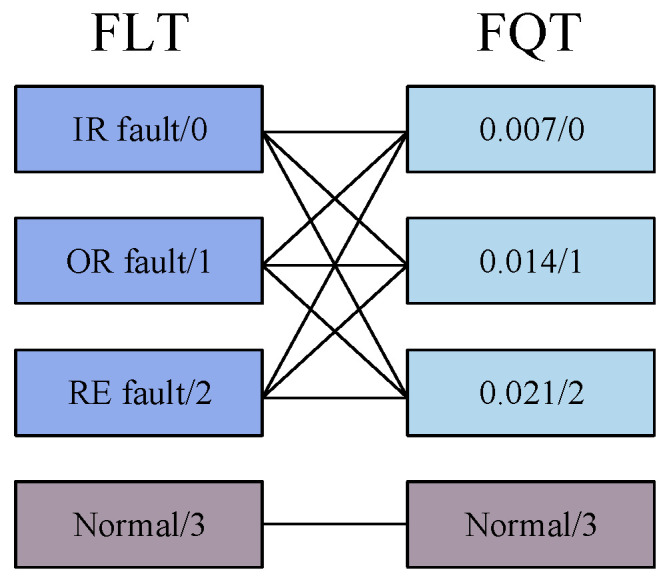
FLT and FQT fault sample interrelationships.

The greater the difference in the distribution of fault features that the model can capture, the better its ability to distinguish between different faults. In other words, the feature distribution of the model can reflect its performance level through T-SNE visualization. According to the visualization results in Figure 6, AMFIS performs best in distinguishing different fault features. In contrast, the feature distributions of WDCNN show significant confounding. Overall, the feature distributions for fault categories 0 to 2 are more severely confounded, which may be due to the high complexity and diversity of fault samples in these categories. For example, the sample of IR faults for FLT covers three different levels of faults, while the sample of 0.007-inch faults for FQT contains faults for IR, OR, and RE. The relationship between these failure types is demonstrated in Figure 7. In contrast, the samples for normal bearings (Label 3) come from a single source and can therefore be effectively differentiated.

**Figure 8:**
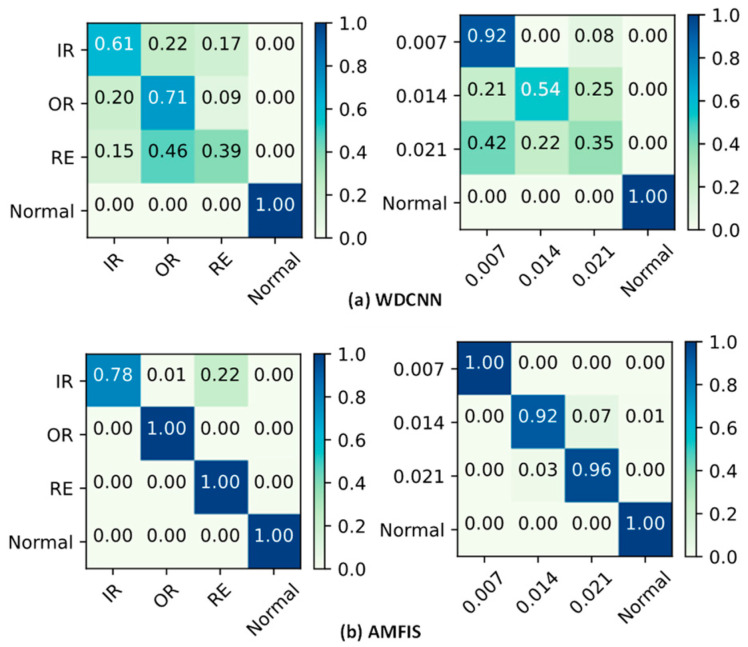
Confusion matrix for diagnostic results.

To further validate the above statement, the ability of WDCNN and AMFIS to identify various fault categories is demonstrated in Figure 8 using a confusion matrix. The results show that WDCNN performs poorly in identifying fault categories 0 to 2, while AMFIS is more effective in identifying various faults. The identification results of AMFIS in Figure 8 match the results of the feature distribution visualization in Figure 6. For example, in the FLT condition, AMFIS tends to misidentify IR as RE, which is consistent with the similarity of the feature distributions for faults 0 and 2 shown in Figure 6.

#### 4.3.3. Performance in Different Noise Environments

**Figure 9:**
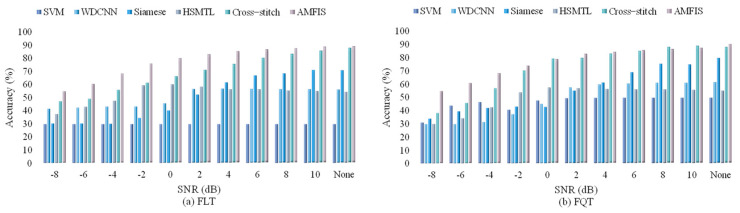
Fault diagnosis results in different noise environments.

In industrial manufacturing, monitoring data is often disturbed by the surrounding environment, which may weaken the ability of trained models to identify faults. The aim of this research section is to evaluate the performance of our proposed method under different noise interference conditions. Consistent with the training process in Section 4.3.2, but prior to the testing phase, we simulate various noisy environments by introducing Gaussian white noise to the test dataset, with Signal-to-Noise Ratios (SNR) settings ranging from −8 dB to 10 dB. It is worth noting that a lower SNR implies a higher energy of the noise. The statistical results of the experiment are detailed in Figure 9.

From Figure 9, we observe that the SVM has no change in FLT under all noises, which indicates that the SVM does not learn efficiently in the FLT of 60 training samples. The performance of the other methods decreases significantly as the SNR decreases. Except for Cross-stitch, which outperforms AMFIS in FQT with SNR = 8 and 10 dB, AMFIS shows the highest performance in both tasks and especially the best performance in FLT. Overall, the performance of the models under different SNR conditions varies significantly, which indicates that SNR has a huge impact on model performance and that different models have different sensitivities to noise.

#### 4.3.4. Performance Under Variable Operating Conditions

In this section, we will explore the performance of the model under variable operating conditions. Variable operating conditions refer to the impact of changes in operating conditions, such as fluctuations in parameters such as speed and load, on monitoring data during industrial production processes. These changes may pose challenges to the stability and accuracy of the model. Therefore, in this section, we explore the performance of various methods under variable operating conditions. The experimental results are shown in Table 4 and Table 5. Among them, 3→0 represents training in 3 hp and testing in 0 hp. Bold is the optimal performance and underlined is the suboptimal performance.

By analyzing the experimental results in Table 4 and Table 5, we can see that there are significant differences in the performance of different algorithms under variable operating conditions. For FLT, AMFIS exhibited the highest performance under almost all testing conditions, particularly reaching 99.33% under 1→3 conditions, demonstrating its strong adaptability and accuracy under variable operating conditions. Cross-stitch closely follows and performs well in most conditions. In contrast, SVM seems unable to handle tasks across different operating conditions, and we repeatedly confirmed that the SVM program did not make any errors. For FQT, AMFIS also maintained the highest performance under most conditions, once again proving its superiority under different operating conditions. The Cross stitch algorithm achieved 93.26% under the condition of 1→2, demonstrating its advantage under specific conditions. The performance of the Siamese algorithm in FQT performance testing is not as good as FLT performance testing, and the overall performance is relatively low. The performance of WDCNN and HSMTL algorithms fluctuates greatly, but they can still demonstrate good performance under certain conditions.

#### 4.3.5. Performance Analysis with Different Number of Training Samples

**Figure 10:**
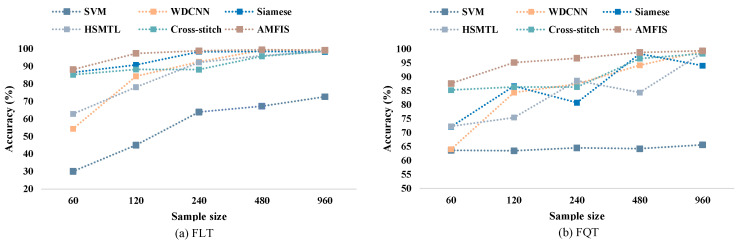
Performance comparison at different training sample sizes.

Currently, the performance of most deep learning-based fault diagnosis models heavily relies on sufficient fault samples, and the number of samples is a key factor affecting the performance of these models. This section aims to evaluate the performance of the proposed method from the perspective of sample size. Specifically, we gradually extract 60 to 960 samples from a dataset with a load of 0 hp for training the models. A summary of the experimental results is presented in Figure 10.

The results in Figure 10 show that the performance of most of the models improves with the increase in the training sample size. However, it is worth noting that the performance of SVM in FQT does not improve with increasing sample size. With less than 480 training samples, AMFIS demonstrates a significant performance advantage. With further increase in sample size, the performance of WDCNN, HSMTL, Siamese, and Cross-stitch models gradually improves and eventually approaches 100% diagnostic accuracy. This phenomenon can be attributed to the fact that the large sample size contains rich fault diagnosis information, which enables these deep learning-based models to learn enough features to achieve satisfactory performance.

### 4.4. Case 2: University of Paderborn Data Set

#### 4.4.1. Introduction to the Dataset and Processing

**Figure 11:**
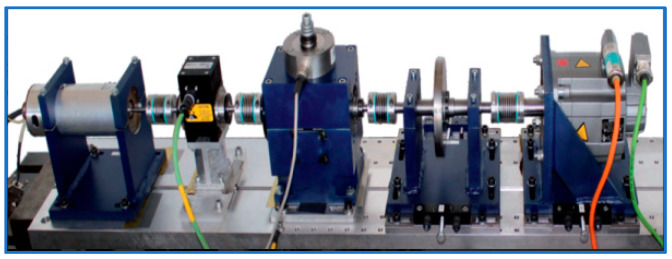
Modular Test bench of PU.

**Figure 12:**
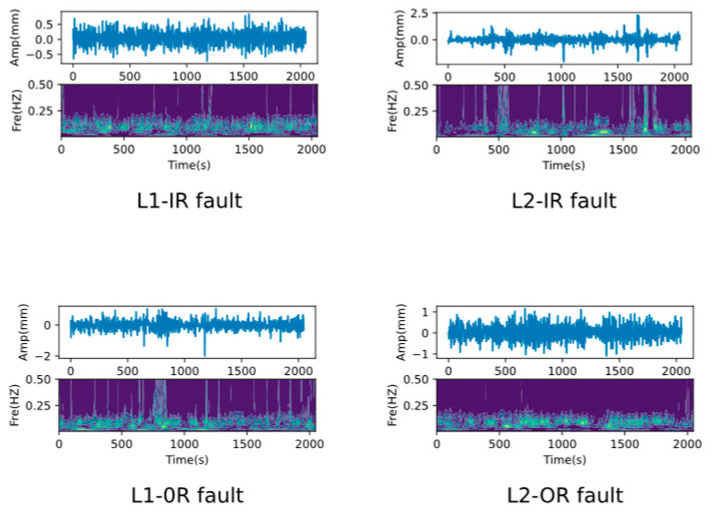
Visualization of the time-frequency diagram for different faults.

The Paderborn University (PU) provided the researchers with a rich bearing dataset on its modular experimental bench, including 6 sets of normal bearing data as well as 12 sets of artificially damaged bearings. The modular lab bench of the PU is shown in Figure 11. These damages include two types of failures, inner and outer rings, and each type has two different levels of failures, level 1 and level 2, and the different failures are time-frequency plots as shown in Figure 12. The sample processing is shown in Figure 5. The specific details of the samples used for the experiments in this paper are in Table 6.

#### 4.4.2. Performance Validation with Small Samples

**Figure 13:**
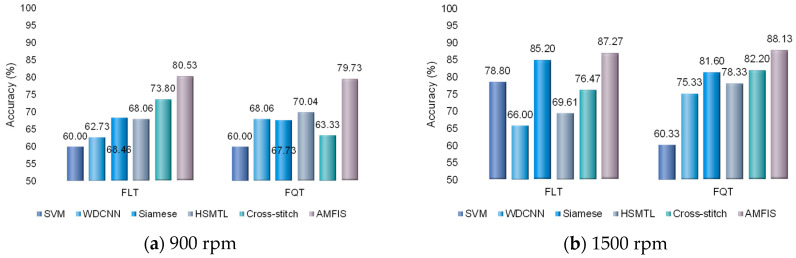
Fault diagnosis results with small samples.

In this section, the main focus is to examine the fault diagnosis performance of the proposed method in the case of small samples. 60 training samples and 2000 test samples are randomly selected. The experimental results are shown in Figure 13.

As can be seen from Figure 13, similar to the results of Case 1 (Section 4.3.2), AMFIS achieves the best results on both FLT and FQT tasks, which shows that AMFIS can share the common knowledge of the two tasks well under small samples to better complete the fault diagnosis task. Siamese’s few-shot learning approach also achieves better results, especially in the 1500 rpm condition. Since Siamese was originally designed to solve the problem of bearing fault diagnosis with small samples, its learning mechanism has advantages in small samples. On the whole, both the soft-shared Cross stitch and hard-shared HSMTL ones also outperform the single-task learning WDCNN and SVM, which proves that MTL is effective in improving the classification performance under small samples by sharing the common knowledge between FLT and FQT. Surprisingly, the SVM achieved excellent results in the FLT task at 1500 rpm, which is significantly different from Case 1.

**Figure 14:**
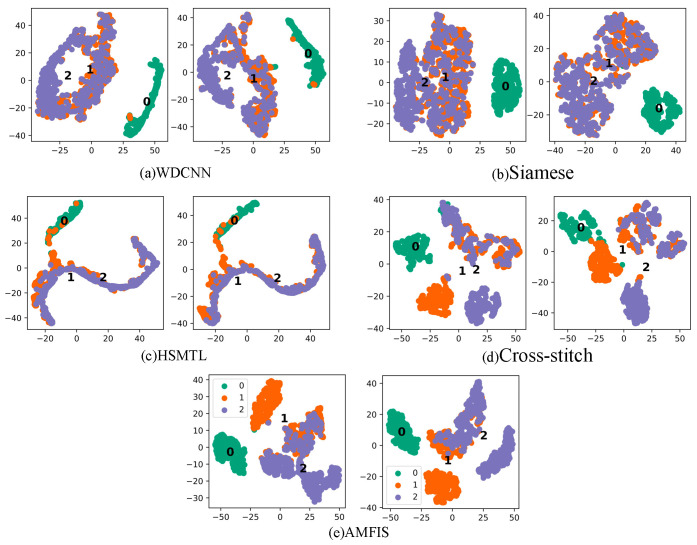
Output layer feature T-SNE visualization. The left image of each set is FLT, and the right image is FQT.

**Figure 15:**
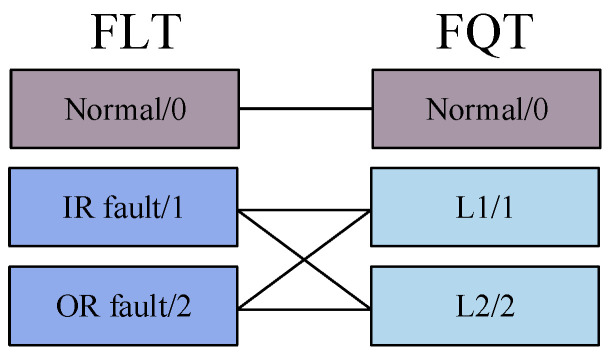
Correlation diagram of fault samples of FLT and FQT for Case 2.

To further report the fault diagnosis results under small samples, the feature distribution of the output layer of the deep learning model was visualized in T-SNE (1500 rpm), as shown in Figure 14. From Figure 14, it can be seen that under small samples, the feature distribution of AMFIS has the highest differentiability, and the model can better distinguish different faults. However, none of the remaining methods can distinguish well between inner and outer circle faults, as well as between level 1 and level 2 fault levels. Similar to Case 1, the actual situation of circle faults and outer ring faults, and level 1 fault degree and level 2 fault degree is more complex, as shown in Figure 15, these methods cannot identify them correctly.

**Figure 16:**
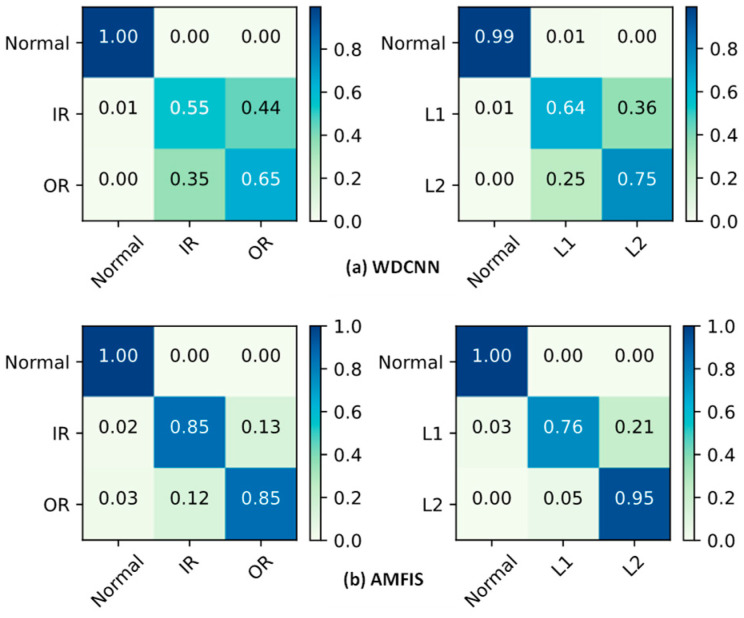
Confusion matrix for diagnostic results.

In order to further observe the recognition performance of each fault category, WDCNN and AMFIS are taken as examples, and their results are presented in a confusion matrix, as shown in Figure 16.

From Figure 15, it can be seen that the model is not good at identifying faults for 1 and 2. However, compared to WDCNN, AMFIS is much better at recognizing 1 and 2 faults.

#### 4.4.3. Performance in Different Noise Environments

**Figure 17:**
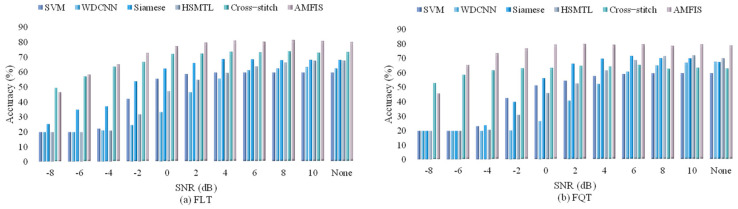
Fault diagnosis results in different noise environments (900 rpm).

We also observe the performance of all methods under different noises in this section, and the experimental process is consistent with Section 4.3.3. The experimental results are shown in Figure 17:

From Figure 9, we observe that, in marked contrast to Case1, SVM gradually improves its performance with increasing signal-to-noise ratio under both tasks. On the whole, the AMFIS model performs optimally under almost all SNR conditions in both the FLT and FQT tasks, while the SVM model performs the worst with lower overall performance in both tasks. AMFIS and Cross-stitch show better performance at low SNR conditions, while some other models show a significant improvement in performance only after the SNR is increased (SNR ≥ −4 dB). have significant improvement. This suggests that different models have different sensitivities to noise, and multi-task learning AMFIS and Cross-stitch have better robustness to noise.

#### 4.4.4. Performance Under Variable Operating Conditions

In this section, we analyze the performance of the model in depth in a variable operating environment. The experimental results are demonstrated in Table 7.

From Table 7, we found that the AMFIS model demonstrated excellent adaptability and stability in both FLT and FQT tasks, especially under conditions of large speed changes, where its performance was most outstanding. The Cross stitch and HSMTL models also demonstrate good generalization ability and can maintain high-performance levels under different speed conditions. In contrast, Siamese, WDCNN, and SVM models exhibit relatively unstable performance under variable operating conditions, while SVM models lag behind in both tasks, indicating their strong dependence on specific operating conditions.

#### 4.4.5. Performance Analysis with Different Number of Training Samples

**Figure 18:**
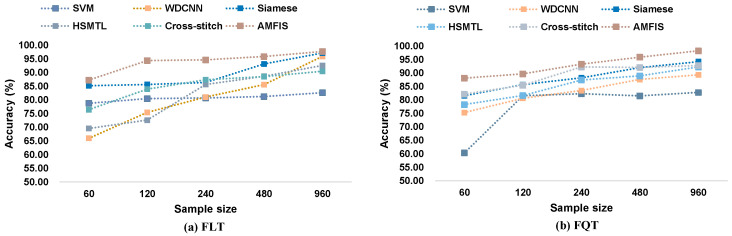
Fault diagnosis results with different training samples.

Like Case 1, this section mainly examines the performance of the proposed method in the PU dataset from the dimension of sample number. Taking 1500 rpm as an example, mainly 60~960 samples are sequentially extracted from the PU dataset for training the model, and the experimental results are shown in Figure 18.

As can be seen in Figure 18, there are some differences with the experimental results of Case 1 in Section 4.3.3. AMFIS achieves the best experimental results with all training samples. Meanwhile, the highest fault classification accuracies of all methods are lower than those of Case 1, indicating that the fault diagnosis task in this case is more complex and requires a larger sample size for training. Overall, AMFIS outperforms other methods by acquiring stronger capabilities when dealing with small-sample tasks through shared knowledge related to different tasks.

### 4.5. Role of the Attention Module

The ability of AM to filter out features from the shared network that are critical to the respective tasks is one of the core designs of AMFIS to implement the MTL sharing mechanism. In this section, this paper validates the effectiveness of the attention module by conducting ablation experiments. In this section, we evaluate the contribution of the attention module by conducting ablation experiments. As an example, the performance of the AMFIS model with and without the attention module (denoted as AMFIS and AMFIS_NAM, respectively) is compared under different load conditions with 60 training samples. The experimental results are summarized and presented in Table 8.

As can be seen from Table 8, AMFIS outperforms AMFIS_NAM in most cases. This suggests that the attention module is able to efficiently filter out features from the shared network that are important for their respective tasks, thus improving the performance of the model. However, there are cases where it is not better than AMFIS_NAM, for example, AMFIS_NAM slightly outperforms AMFIS on the 0 hp and 1 hp FQT tasks.

### 4.6. Role Analysis of DAS

In the optimization process of MTL, the choice of optimization intensity for different tasks is crucial because of their different learning difficulties. In this paper, a DAS algorithm is proposed to adjust the optimization intensity of FLT and FQT in real-time. The aim of this section is to analyze the effectiveness of the DAS algorithm in the model optimization process by performing ablation experiments to compare the model performance with and without the DAS algorithm. The experimental results are summarized and presented in Table 9, where AMFIS_NDAS is dynamically tuned without DAS, i.e., $w_{i}\left(t\right)$ is always 1 during the optimization process.

**Figure 19:**
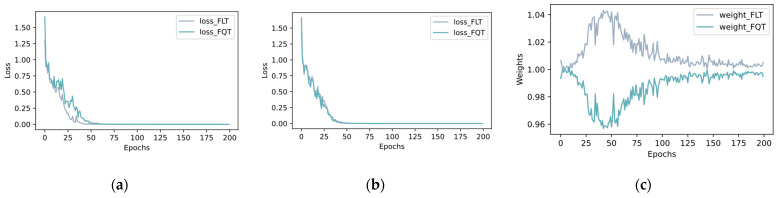
The loss changes curves of FLT and FQT during training, and the weight changes: (**a**) AMFIS_NDAS loss; (**b**) AMFIS loss; (**c**) AMFIS weight changes.

From Table 9, it can be seen that DAS can bring small gains to AMFIS in most cases, verifying the effectiveness of the DAS algorithm for the optimization process. In order to further understand the effectiveness of DAS, $w_{1}\left(t\right)$ and $w_{2}\left(t\right)$ of the training process, as well as the losses of FLT and FQT, are recorded in this paper and presented in Figure 19.

### 4.7. Other Analysis

#### 4.7.1. The Inference Speed of the Model

**Figure 20:**
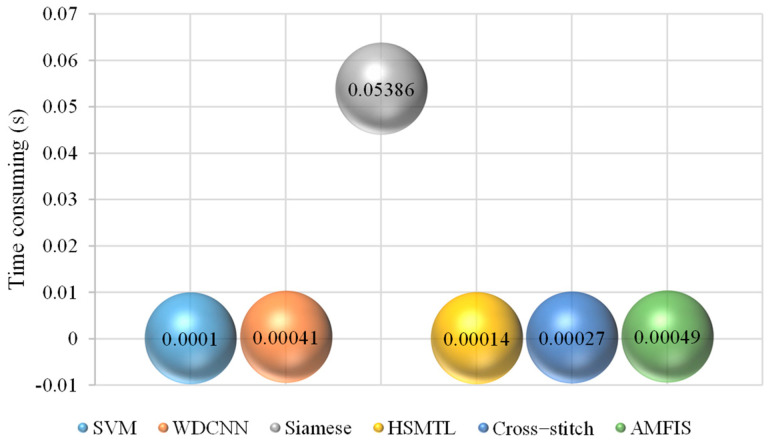
Time consumed by model inference.

In modern industry and intelligent manufacturing, the inference speed of the fault diagnosis model is one of the key indexes to measure its performance. The inference speed is directly related to the timeliness and effectiveness of fault diagnosis, which is shown to be of great significance for ensuring production safety and improving production efficiency. In this section, experiments are conducted on the inference speed of the model (the experimental environment is described in Section 3.1), and Figure 20 shows the time taken by the model to diagnose one sample.

According to the data in Figure 16, the Siamese model has the longest testing time of 0.05386 s, which is significantly higher than the other models. This phenomenon can be attributed to the fact that the Siamese model employs an N-way k-shot strategy in the testing phase, which requires a comparison with every sample in the support set, leading to a higher computation time. In contrast, the testing time of the AMFIS model is only 0.00049 s, a value that is much lower than the sampling time required in engineering applications and, therefore, of high value in practical applications.

#### 4.7.2. Preliminary Validation Extended to 3 Tasks

AMFIS is easily scalable to more tasks, and we are curious about its performance when scaling to more tasks. To this end, we conducted a preliminary experimental study. Taking the 1500 rpm working condition in Case 2 as an example, we further added a Fault Detection Task (FDT) to the existing FLT and FQT. The core objective of this new task is to diagnose whether the equipment is malfunctioning or not. The experimental results of the two tasks and the three tasks are shown in Table 10.

From Table 10, it can be seen that AMFIS, as a multi-task learning framework, has a performance of 87.27% on FLT and 88.13% on FQT when dealing with 2 tasks. When the number of tasks is extended to 3, AMFIS achieves 98.79% accuracy on FDT, and its performance on FLT and FQT tasks is also improved to 91.56% and 89.17%, respectively. The results of this preliminary experiment suggest that AMFIS can effectively benefit from multi-task learning, where knowledge learned on one task is utilized by other tasks, thus improving the generalization of the model. However, specific conclusions need to be validated by experiments with more suitable cases. In the future, we will look for more suitable cases to test the performance of AMFIS in more tasks.

## 5. Conclusions

Bearing fault diagnosis in real engineering is often a typical small-sample problem, and it is of great practical significance to acquire fault information in multiple dimensions simultaneously. In order to acquire sufficient fault diagnosis knowledge under small samples and achieve the tasks of fault localization and fault quantification at the same time, a multidimensional fault information sharing method based on AM is proposed (AMFIS), where AMFIS can share the common knowledge between the fault localization and quantification tasks to improve the performance of all tasks under small samples. Meanwhile, in order to ensure that both FLT and FQT tasks can achieve optimal training results, this paper proposes an innovative dynamic adjustment strategy, which can adaptively adjust the training weights of FLT and FQT tasks according to the training process to achieve more effective task optimization.

Extensive experiments were conducted on two different case datasets, and the results confirmed that AMFIS effectively enriches the knowledge of fault diagnosis under small-sample conditions through its task knowledge-sharing mechanism and is able to excel in small-sample fault diagnosis tasks. Compared with some mainstream methods, AMFIS demonstrates a higher fault diagnosis capability in small-sample environments. In addition, the effectiveness of the attention module and DAS optimization settings in AMFIS is further verified through ablation experiments.

However, there are still many challenges to overcome when implementing our method in practice, such as the generalizability of performance under complex and variable operating conditions and the ability to identify unknown new faults.

## Figures and Tables

**Table 1:** Comparison methods.

Methods	Introduce
SVM	Classical machine learning methods whose performance we are curious about.
WDCNN	The classical single-task fault diagnosis method. This is a CNN model with a wide kernel in the first layer, which is set up in such a way as to extract short-time features that act similarly to the short-time Fourier transform.
Siamese	This method uses a unique few-shot learning strategy, which is a specialized method for diagnosing bearing faults in small samples.
HSMTL	A hard-shared multi-task learning approach based on WDCNN encoders. In this method, multiple tasks use a common shallow encoder, but the output layer is set independently for each task.
Cross-stitch	Advanced soft-shared MTL method. The method is used to dynamically adjust the proportion of feature fusion from different tasks by learning a set of weights.
AMFIS	The proposed methodology.

**Table 2:** Training sample information of Case 1.

Load (hp)	Sample Size (Number)		Category/Label	
	Training	Testing	FLT	FQT
0	60 120 240 480 960	2000	IR/0 OR/1 RE/2 Normal/3	0.007/0 0.014/1 0.021/2 Normal/3
1				
2				
3				

**Table 3:** Fault diagnosis results for small samples.

Methods	SVM		WDCNN		Siamese		HSMTL		Cross-Stitch		AMFIS	
Tasks	FLT	FQT	FLT	FQT	FLT	FQT	FLT	FQT	FLT	FQT	FLT	FQT
0 hp	30.03	63.70	54.33	64.03	86.46	72.13	62.83	72.26	85.23	85.26	**88.13**	**87.63**
1 hp	30.06	50.03	53.13	61.46	68.08	**84.43**	54.49	59.36	85.66	83.27	**89.77**	84.17
2 hp	30.03	50.13	56.63	58.56	72.80	82.89	64.23	60.55	86.45	88.66	**89.97**	**90.33**
3 hp	30.00	50.20	71.43	66.50	71.63	86.03	59.53	61.79	89.43	90.90	**92.87**	**95.67**

**Table 4:** FLT performance under variable operating conditions.

	3→0	0→3	3→1	1→3	3→2	2→3	2→0	0→2	2→1	1→2	1→0	0→1
SVM	30.00	30.00	30.00	30.00	30.00	30.00	30.00	30.00	30.00	30.00	30.00	30.06
WDCNN	34.41	52.85	58.37	49.17	56.45	57.87	38.72	46.51	49.10	52.65	40.23	50.39
Siamese	50.14	63.16	81.07	68.63	72.61	72.40	55.92	66.46	57.07	68.38	52.19	66.43
HSMTL	36.91	41.81	54.96	50.28	51.99	58.03	38.19	44.76	50.16	53.50	40.61	43.50
Cross-stitch	65.23	62.70	**89.77**	94.50	90.31	91.86	67.80	**84.00**	93.28	92.20	66.38	**76.27**
AMFIS	**69.12**	**83.16**	88.85	**99.33**	**91.56**	**93.66**	**70.40**	82.56	**94.87**	**93.36**	**69.62**	75.64

**Table 5:** FQT performance under variable operating conditions.

	3→0	0→3	3→1	1→3	3→2	2→3	2→0	0→2	2→1	1→2	1→0	0→1
SVM	50.00	49.90	50.00	50.00	50.00	50.00	50.00	48.16	50.00	50.00	49.83	46.40
WDCNN	48.80	50.50	58.76	60.31	63.55	61.84	47.65	51.04	59.98	60.85	44.68	51.84
Siamese	37.02	37.54	42.26	41.48	33.96	42.74	32.95	30.76	42.77	40.81	34.21	36.43
HSMTL	46.69	49.55	56.48	58.24	61.71	61.56	43.19	49.34	57.37	59.12	43.29	46.18
Cross-stitch	60.40	47.76	85.05	75.20	82.15	83.15	58.77	55.06	81.94	**93.26**	57.97	58.68
AMFIS	**69.46**	**63.66**	**87.87**	**89.73**	**87.26**	**92.66**	**63.42**	**58.06**	**87.50**	92.46	**61.39**	**62.12**

**Table 6:** Training sample information of Case 2.

Code	Rotational Speed (rpm)	Fault (Location/Degree)	Sample Size (Number)		Category/Label	
			Training	Testing	FLT	FQT
KA_	900 1500	OR/L2	60 120 240 480 960	2000	OR/2 IR/1 Normal/0	L2/2 L1/1 Normal/0
		OR/L1				
KI_		IR/L2				
		IR/L1				
K0_		Normal				

**Table 7:** Experimental results under variable speed conditions.

	900 rpm–1500 rpm		1500 rpm–900 rpm	
	FLT	FQT	FLT	FQT
SVM	21.20	25.06	40.26	45.60
WDCNN	38.69	31.93	51.36	49.65
Siamese	50.92	22.16	57.02	52.58
HSMTL	42.96	44.14	55.65	54.51
Cross-stitch	52.09 *	53.19	52.44	51.33
AMFIS	**59.53**	**54.33**	**58.86**	**59.58**

* The bolded numbers represent the best performance, while the underlined numbers indicate the sub-optimal performance.

**Table 8:** Experimental results with and without AM.

Methods		AMFIS_NAM		AMFIS	
Tasks		FLT	FQT	FLT	FQT
Case 1	0 hp	87.27	**88.70** *	**88.13**	87.63
	1 hp	89.63	**86.03**	**89.77**	84.17
	2 hp	88.26	86.25	**89.97**	**90.33**
	3 hp	92.39	88.83	**92.87**	**95.67**
Case 2	900 rpm	78.55	75.66	**80.53**	**79.73**
	1500 rpm	86.33	86.33	**87.27**	**88.13**

* The bolded numbers represent the best performance.

**Table 9:** Experimental results with and without DAS.

Methods		AMFIS_NDAS		AMFIS	
Tasks		FLT	FQT	FLT	FQT
Case 1	0 hp	86.57	86.83	**88.13** *	**87.63**
	1 hp	84.63	**86.70**	**89.77**	84.17
	2 hp	88.63	86.40	**89.97**	**90.33**
	3 hp	92.56	93.69	**92.87**	**95.67**
Case 2	900 rpm	78.01	75.36	**80.53**	**79.73**
	1500 rpm	86.23	86.07	**87.27**	**88.13**

* The bolded numbers represent the best performance.

**Table 10:** AMFIS performance in a wider range of tasks.

	FDT	FLT	FQT
2 tasks	\	87.27	88.13
3 tasks	***98.79*** *	***91.56***	***89.17***

* The bolded numbers represent the best performance.

## Data Availability

We can assist in providing AMFIS codes and data upon reasonable request.

## References

[1] Gao S., Xu L., Zhang Y., Pei Z. (2022). Rolling Bearing Fault Diagnosis Based on SSA Optimized Self-Adaptive DBN. ISA Trans..

[2] Tahmasbi D., Shirali H., Sajad Mousavi Nejad Souq S., Eslampanah M. (2024). Diagnosis and Root Cause Analysis of Bearing Failure Using Vibration Analysis Techniques. Eng. Fail. Anal..

[3] Liao Y., Huang R., Li J., Chen Z., Li W. (2021). Dynamic Distribution Adaptation Based Transfer Network for Cross Domain Bearing Fault Diagnosis. Chin. J. Mech. Eng..

[4] Wang C., Chen X., Qiang X., Fan H., Li S., Wang C., Chen X., Qiang X., Fan H., Li S. (2024). Recent Advances in Mechanism/Data-Driven Fault Diagnosis of Complex Engineering Systems with Uncertainties. AIMS Math..

[5] LeCun Y., Bengio Y., Hinton G. (2015). Deep Learning. Nature.

[6] Fu G., Wei Q., Yang Y. (2024). Bearing Fault Diagnosis with Parallel CNN and LSTM. Math. Biosci. Eng..

[7] Li Y., Zhang Y., Wang R., Fu J. (2024). A Reinforcement Ensemble Learning Method for Rolling Bearing Fault Diagnosis Under Variable Work Conditions. Sensors.

[8] Lei X., Lu N., Chen C., Wang C. (2022). An AVMD-DBN-ELM Model for Bearing Fault Diagnosis. Sensors.

[9] Jia H.E. (2024). Train Bearing Fault Diagnosis Based on Convolution Temporal-Spatial Mutual Fusion Network. Chengshi Guid. Jiaotong Yanjiu.

[10] Wu J., Zhao Z., Sun C., Yan R., Chen X. (2020). Few-Shot Transfer Learning for Intelligent Fault Diagnosis of Machine. Measurement.

[11] Lee D., Jeong J. (2023). Few-Shot Learning-Based Light-Weight WDCNN Model for Bearing Fault Diagnosis in Siamese Network. Sensors.

[12] Wang S., Wang D., Kong D., Wang J., Li W., Zhou S. (2020). Few-Shot Rolling Bearing Fault Diagnosis with Metric-Based Meta Learning. Sensors.

[13] Li C., Li S., Feng Y., Gryllias K., Gu F., Pecht M. (2024). Small Data Challenges for Intelligent Prognostics and Health Management: A Review. Artif. Intell. Rev..

[14] Li C., Luo K., Yang L., Li S., Wang H., Zhang X., Liao Z. (2024). A Zero-Shot Fault Detection Method for UAV Sensors Based on a Novel CVAE-GAN Model. IEEE Sens. J..

[15] Liang X., Zhang M., Feng G., Wang D., Xu Y., Gu F. (2023). Few-Shot Learning Approaches for Fault Diagnosis Using Vibration Data: A Comprehensive Review. Sustainability.

[16] Xin R., Feng X., Wang T., Miao F., Yu C. (2023). A Multi-Task-Based Deep Multi-Scale Information Fusion Method for Intelligent Diagnosis of Bearing Faults. Machines.

[17] Zhang Y., Yang Q. (2022). A Survey on Multi-Task Learning. IEEE Trans. Knowl. Data Eng..

[18] Zhang Z., Yu W., Yu M., Guo Z., Jiang M. (2023). A Survey of Multi-Task Learning in Natural Language Processing: Regarding Task Relatedness and Training Methods. Proceedings of the 17th Conference of the European Chapter of the Association for Computational Linguistics.

[19] Sosnin S., Vashurina M., Withnall M., Karpov P., Fedorov M., Tetko I.V. (2019). A Survey of Multi-Task Learning Methods in Chemoinformatics. Mol. Inform..

[20] Zhang J., Chen J., Deng H., Hu W. (2023). A Novel Framework Based on Adaptive Multi-Task Learning for Bearing Fault Diagnosis. Energy Rep..

[21] Chen L., Xu G., Tao T., Wu Q. (2020). Deep Residual Network for Identifying Bearing Fault Location and Fault Severity Concurrently. IEEE Access.

[22] Li J., Wei Y., Gu X. (2024). MTC-GAN Bearing Fault Diagnosis for Small Samples and Variable Operating Conditions. Appl. Sci..

[23] Zhang Y., Li S., Yang L., Zhu Y., Liao Z., Wang Y., Li C., Wang H. (2024). FW-UAV Fault Diagnosis Based on Multilevel Task Knowledge Supplement Network Under Small Samples. IEEE Trans. Instrum. Meas..

[24] Wang H., Liu Z., Peng D., Yang M., Qin Y. (2022). Feature-Level Attention-Guided Multitask CNN for Fault Diagnosis and Working Conditions Identification of Rolling Bearing. IEEE Trans. Neural Netw. Learn. Syst..

[25] Vandenhende S., Georgoulis S., Van Gansbeke W., Proesmans M., Dai D., Van Gool L. (2022). Multi-Task Learning for Dense Prediction Tasks: A Survey. IEEE Trans. Pattern Anal. Mach. Intell..

[26] Wang X., Yu C., Gu Y., Hu M., Ren F. (2021). Multi-Task and Attention Collaborative Network for Facial Emotion Recognition. IEEJ Trans. Electr. Electron. Eng..

[27] Zhang Y., Li S., Zhang A., An X. (2024). FW-UAV Fault Diagnosis Based on Knowledge Complementary Network under Small Sample. Mech. Syst. Signal Process..

[28] Vaswani A., Shazeer N., Parmar N., Uszkoreit J., Jones L., Gomez A.N., Kaiser Ł., Polosukhin I. Attention Is All You Need. Proceedings of the Advances in Neural Information Processing Systems.

[29] Liu X., Tang H., Zhao J., Dou Q., Lu M. (2023). TCAMixer: A Lightweight Mixer Based on a Novel Triple Concepts Attention Mechanism for NLP. Eng. Appl. Artif. Intell..

[30] Guo M.-H., Xu T.-X., Liu J.-J., Liu Z.-N., Jiang P.-T., Mu T.-J., Zhang S.-H., Martin R.R., Cheng M.-M., Hu S.-M. (2022). Attention Mechanisms in Computer Vision: A Survey. Comp. Vis. Media.

[31] Hu J., Shen L., Sun G. Squeeze-and-Excitation Networks. Proceedings of the 2018 IEEE Conference on Computer Vision and Pattern Recognition.

[32] Woo S., Park J., Lee J.-Y., Kweon I.S. CBAM: Convolutional Block Attention Module. Proceedings of the 15th European Conference on Computer Vision (ECCV).

[33] Wang Q., Wu B., Zhu P., Li P., Zuo W., Hu Q. ECA-Net: Efficient Channel Attention for Deep Convolutional Neural Networks. Proceedings of the 2020 IEEE/CVF Conference on Computer Vision and Pattern Recognition.

[34] Zhang X., He C., Lu Y., Chen B., Zhu L., Zhang L. (2022). Fault Diagnosis for Small Samples Based on Attention Mechanism. Measurement.

[35] Hu Z., Zhang X., Xiong H. (2024). Two-Stage Attention Network for Fault Diagnosis and Retrieval of Fault Logs. Expert Syst. Appl..

[36] Yang Z., Zhang J., Zhao Z., Zhai Z., Chen X. (2020). Interpreting Network Knowledge with Attention Mechanism for Bearing Fault Diagnosis. Appl. Soft Comput..

[37] Zhang W., Peng G., Li C., Chen Y., Zhang Z. (2017). A New Deep Learning Model for Fault Diagnosis with Good Anti-Noise and Domain Adaptation Ability on Raw Vibration Signals. Sensors.

[38] Misra I., Shrivastava A., Gupta A., Hebert M. Cross-Stitch Networks for Multi-Task Learning. Proceedings of the 2016 IEEE Conference on Computer Vision and Pattern Recognition.

